# Association of lipid accumulation product and visceral adiposity index with the risk of hypertension among oil workers in Xinjiang, China

**DOI:** 10.7717/peerj.15273

**Published:** 2023-05-17

**Authors:** Guliman Muheiyati, Yujie Mei, Ning Tao

**Affiliations:** 1 School of Public Health, Xinjiang Medical University, Urumqi, Xinjiang, China; 2 Xinjiang Clinical Research Center for Genitourinary System, Xinjiang Medical University, Urumqi, Xinjiang, China

**Keywords:** Hypertension, Visceral adiposity index, Restricted cubic spline, Lipid accumulation product, Oil workers

## Abstract

**Background:**

To explore the relationship between lipid accumulation product (LAP) and visceral adiposity index (VAI) and hypertension in oil workers and to evaluate the predictive value of hypertension by gender.

**Methods:**

A sample of 2,312 workers aged 18–60 years old with more than one year of service were selected by a whole-group random sampling method in six oil field bases in Karamay City, Xinjiang. Logistic regression combined with restricted cubic spline model was used to analyze the risk of hypertension in different LAP and VAI. The receiver operator characteristic curve (ROC) with different sex LAP and VAI predicting the risk of hypertension were drawn.

**Results:**

(1) There were significant differences in age, smoking, alcohol consumption, hypertension, BMI, WC, WHtR, SBP, DBP, TC, TG, HDL, LDL, FPG and Scr among different gender groups (*P* < 0.001).The prevalence of hypertension was 10.1%, with 13.9% in men and 3.6% in women. The prevalence of hypertension with different individual characteristics was statistically significant (*P* < 0.05). (2) Lipid accumulation product and visceral adiposity index were positively associated with hypertension (*P* < 0.001). The risk of hypertension may increase with the increase of lipid accumulation product and visceral adiposity index. After adjusting for age, sex, BMI, Scr, FPG and other factors, the risk of hypertension in the fourth quartile was (OR = 5.69, 95% CI [2.72–11.8]) and (OR = 3.56, 95% CI [2.03–6.23]) compared with the first quartile of lipid accumulation product and visceral adiposity index. (3) ROC results showed: AUC values of 0.658 (95% CI [0.619–0.696]), 0.614 (95% CI [0.574–0.654]), 0.661 (95% CI [0.620–0.703]) and critical values of 43.25, 1.58, 0.13 for LAP, VAI and combined indicators in men; the AUC values of LAP, VAI and combined indicators for women were 0.787 (95% CI [0.710–0.865]), 0.732 (95% CI [0.640–0.825]), 0.792 (95% CI [0.719–0.864]) and the critical values were 35.73, 1.76 and 0.03. Restricted cubic splines showed a nonlinear dose-response relationship between LAP, VAI, and risk of hypertension prevalence (*P* < 0.01 for overall trend and *P* < 0.01 for nonlinearity).

**Conclusions:**

Lipid accumulation product and visceral adiposity index may be risk factors for hypertension in oil workers. LAP and VAI have certain predictive value for hypertension.

## Introduction

Hypertension is the most serious public health problem and poses a serious threat to human health (NCD Risk Factor Collaboration, 2021). Hypertension is a major risk factor for cardiovascular disease (Huang et al., 2019). Surveys show that the incidence of hypertension exceeds 40% among adults over 25 years of age worldwide, creating a heavy global burden of disease (Wang et al., 2018). In addition, recent studies have shown that obesity (Feng et al., 2016; Zhu et al., 2021), especially visceral obesity (Chung et al., 2017; Zhang et al., 2022), is closely associated with hypertension. Waist circumference (WC), body mass index (BMI), waist-to-hip ratio (WHR) and waist-to-height ratio (WHtR) are often used as assessment indicators to define obesity, but these indicators do not accurately reflect body fat content, distribution and function (Zhang et al., 2013; Pekgor et al., 2019). At present, there are computed tomography, magnetic resonance imaging and dual-energy X-ray absorptiometry can quantify the content of human adipose tissue (Demirbas & Kutlu, 2021). However, visceral fat function cannot be accurately evaluated, and factors such as radiation exposure, high cost and time consuming are considered. The distribution of hypertensive patients in China is mostly in rural areas, and there are medical environments and economic levels that are not suitable for widespread promotion and use in clinical practice. In recent years, researchers have proposed two new body fat indices, the lipid accumulation product (LAP) and visceral adiposity index (VAI), which can be used as evaluation parameters for excessive lipid accumulation in humans (Amato et al., 2010). Lipid accumulation product (LAP) was proposed by Kahn (2006) and is considered to be a relatively accurate indicator of the degree of lipid accumulation and visceral adiposity in humans. Studies have shown that both LAP and VAI can be used as effective indicators to identify visceral obesity (Roriz et al., 2014). Compared with typical body fat indicators, they can better predict the occurrence of cardiovascular disease. oil workers are a special occupational group, as oil operations are mostly located in the Gobi Desert far from urban areas and work shifts, there are higher rates of unhealthy behaviors such as drinking and smoking, which increase the risk of hypertension (Yang et al., 2021; An et al., 2021; Li et al., 2019). Underestimating the risk of hypertension leads to missed opportunities for treatment and prevention. especially in young adults, can be effective in reducing the risk of hypertension progression and cardiovascular disease. The high risk of hypertension leads to additional costs, unnecessary treatment and potential adverse treatment effects. At present, there are few studies related to LAP, VAI and hypertension in occupational groups at home and abroad, and their predictive value needs to be further evaluated. Therefore, this study aims to investigate the relationship between LAP, VAI and hypertension in this occupational group of oil workers, which is of great practical importance.

## Materials and Methods

### Study subjects

Using the whole-group random sampling method, a total of 2,312 employees aged 18–65 years old who participated in health checkups were randomly selected from four districts in Karamay City, Xinjiang, with operational units as clusters and six randomly selected oilfield enterprises and service units from April to June 2021. The examinees included oil recovery, gathering workers, thermal operators, painters, welders, drilling diesel engine workers, and chemical process testers. After excluding 126 of them with incomplete medical examination information, 2,186 subjects were finally included in the study. Inclusion criteria: (1) those with complete physical examination data related to this study; (2) those who were willing to cooperate with this investigation and signed the informed consent form. Exclusion criteria: (1) Patients with serious organic lesions, mental illnesses, and genetic diseases; (2) Those with incomplete physical examination data. The sample content estimation formula is: *N* = μ^2^_α_ ρ( 1 − ρ)/δ^2^, the pre-survey showed that the prevalence of hypertension ρ = 0.16, α = 0.05 (bilateral), and the tolerance error δ = 0.02. *N* = 1,291 was calculated, taking into account a 20% loss, at least 1,549 cases need to be included in the study, and the number of people surveyed in this current study is 2,186. The sample size requirement was satisfied. All participants signed a written informed consent. The study was approved by the Ethics Review Committee of the First Affiliated Hospital of Xinjiang Medical University (No. 2015006).

## Methods

### Anthropometric and defintion

The general survey and physical examination information collected by the staff of the physical examination section of the central hospital in Karamay. The general conditions mainly include age, gender, alcohol consumption, smoking, and history of previous diseases. According to the relevant definition of WHO, Smoker: smoking ≥1 cigarette per day for 6 months or more; drinker: drinking ≥1 times a week with alcohol intake ≥50 g per drinking session regularly ≥1 year. The physical examination is done by the professional staff of the Physical Examination Center of Karamay Central Hospital, which mainly includes the measurement of height, weight, waist circumference, blood pressure, blood glucose and lipid, and the calculation of BMI, WHtR, LAP and VAI. Height and weight measurement by automatic recorder (SK-X80/TCS-160D-W/h) standard position without shoes. After the subject rested for 5 min, the blood pressure was measured in the sitting arm using an Omron (HEM-7211; Omron, Kyoto, Japan) electronic blood pressure monitor, and the average value was taken after three measurements. A fully automated biochemical analyzer (Beckman Coulter AU5800; Beckman Coulter, Brea, CA, USA) was used for biochemical analysis, detection of TG, TC, HDL-C, LDL-C, FPG and serum creatinine (Scr).

### Blood pressure measurement and blood biochemical assays

Hypertension if they met one of the following standards (Joint Committee for Guideline Revision, 2019): (1) systolic blood pressure ≥ 140 mm Hg and/or diastolic blood pressure ≥ 90 mm Hg; (2) self-reported hypertension diagnosed by a physician and current antihypertensive treatment during the previous 2 weeks (China Hypertension Prevention and Control Guidelines Revision Committee, Hypertension Alliance China, Chinese Society of Cardiovascular Diseases Chinese Physicians Association Hypertension Professional Committee, 2019). ① BMI < 18.5 kg/m^2^ is too low body weight (BW), BMI between 18.5 and 23.9 kg/m^2^ is normal BW, BMI between 24.0 and 27.9 kg/m^2^ is overweight, and BMI ≥ 28 kg/m^2^ is obese (China Working Group on Obesity, 2004). ② WC ≥ 90 cm in men and 85 cm in women is considered centrally obese (Bao et al., 2008). ③ central obesity when WHtR ≥ 0.5.

### Calculation of related indices

1. BMI was calculated as:

$\rm {BMI = BW\,(kg)/body\, height\,(BH^2)\,(m)}$ (China Working Group on Obesity, 2004).

2. WHtR was calculated as:

$\rm {WHtR = WC\, (cm)/BH\, (cm)}$ (Browning, Hsieh & Ashwell, 2010).

3. LAP was calculated as:

$\rm {LAP = (WC\,(cm) - 65) \times TG\,(mmol/L) )}$ in males, and

${\rm{LAP}} = ({\rm{WC}}{\mkern 1mu} ({\rm{cm}}) - 58) \times {\rm{TG}}{\mkern 1mu} ({\rm{mmol}}/{\rm{L}}){\mkern 1mu} {\rm{in}}{\mkern 1mu} {\rm{females}}$ (Kahn, 2006).

4. VAI score was calculated as described previously (Amato et al., 2010) using the following sex-specific equations (with TG levels in mmol/L and HDL-cholesterol levels in mmol/L):

${\rm{VAI = }}\left( {\matrix{
   {{{{\rm{WC}}\left( {{\rm{cm}}} \right)} \over {39.68 + \left( {1.88 \times {\rm{BMI}}} \right)}}\, \times \,} & {{{{\rm{TG(mmol/I)}}} \over {1.03}}\, \times \,} & {{{{\rm{1}}{\rm{.31}}} \over {{\rm{HDL(mmol/I)}}}}}  \cr 

 } } \right)$ in males,

${\rm{VAI = }}\left( {\matrix{
   {{{{\rm{WC}}\left( {{\rm{cm}}} \right)} \over {36.58 + \left( {1.89 \times {\rm{BMI}}} \right)}}\, \times \,} & {{{{\rm{TG(mmol/I)}}} \over {0.81}}\, \times \,} & {{{{\rm{1}}{\rm{.52}}} \over {{\rm{HDL(mmol/I)}}}}}  \cr 

 } } \right)$ in females.

### Statistical analysis

Analyses were performed using SPSS Version 26.0 (IBM, Chicago, IL, USA) and R version 4.0.5 data were expressed as mean ± standard deviation for normally distributed continuous variables and median (*P*_25_, *P*_75_) for not normally distributed continuous variables, and the t-test or non-parametric test was used to compare the two groups. Count data were expressed as rates or composition ratios and data were compared using chi-square tests. Spearman Rank correlation was used for correlation analysis. LAP and VAI indices were grouped using the quartile method (LAP: “Q1” is ≤12.8, “Q2” is 12.8–27.51, “Q3” is 27.51–50.71, “Q4” is >50.71; VAI: “Q1” is ≤0.86, “Q2” is 0.86–14.6, “Q3” is 14.6–2.39, “Q4” is >2.39). The dose-response relationship between LAP and VAI index and hypertension was analyzed by using restricted cubic spline and logistic regression model. The test level was α = 0.05.

## Results

### Baseline characteristics of oil workers

A total of 2,186 persons were included, 1,372 of whom were men, aged (41.58 ± 10.03) years. There were 814 females with an age of (39.59 ± 9.00) years. Statistically significant differences were found in the comparison of age, history of alcohol consumption, history of smoking, hypertension, BMI, WC, WHtR, SBP, DBP, TC, TG, HDL-C, LDL-C, FPG, Scr, LAP and VAI in different gender groups (*P* < 0.01) (Table 1).

**Table 1:** Baseline characteristics of oil workers.

Variables	Male (*N* = 1,372)	Female (*N* = 814)	*t/* ${\chi ^2}$ */z*	*P-*value
Age (years)	41.58 $\pm$ 10.03	39.59 $\pm$ 9.00	4.654	<0.001
Smoking (cases (%))	644 (46.9)	2 (0.2)	535	<0.001
Drinking (cases (%))	768 (56.0)	24 (2.9)	621	<0.001
Hypertension (cases (%))	191 (13.9)	29 (3.6)	60.566	<0.001
BMI (kg/m^2^)	25.4 (23.4, 27.7)	22.3 (20.2, 24.8)	−18.927	<0.001
WC (cm)	89.85 $\pm$ 9.99	75.96 $\pm$ 9.56	31.928	<0.001
WHtR	0.52 $\pm$ 0.06	0.47 $\pm$ 0.06	19.113	<0.001
SBP (mmHg)	128.98 $\pm$ 22.39	115.69 $\pm$ 14.57	15.141	0.001
DBP (mmHg)	82.59 $\pm$ 11.79	72.85 $\pm$ 9.76	19.864	0.001
TG (mmol/L)	1.53 (1.01, 2.27)	0.89 (0.67, 1.27)	−19.634	<0.001
TC (mmol/L)	5.17 (4.58, 5.86)	5.03 (4.44, 5.66)	−3.775	<0.001
HDL-C (mmol/L)	1.15 (0.99, 1.33)	1.44 (1.24, 1.67)	−20.861	<0.001
LDL-C (mmol/L)	2.90 (2.45, 3.46)	2.62 (2.17, 3.10)	−8.389	<0.001
FPG (mmol/L)	5.28 (4.96, 5.72)	5.11 (4.83, 5.42)	−8.053	<0.001
Scr (μmol/L)	81.65 $\pm$ 17.60	60.40 $\pm$ 8.92	32.086	0.001
LAP	37.16 (20.42, 65.15)	14.35 (7.82, 27.9)	−20.666	<0.001
VAI	1.74 (0.99, 2.81)	1.09 (0.71, 1.73)	−13.272	0.001

**Note:**

Age, WC, WHtR, SBP, DBP, and Scr are expressed as (x $\pm$ s), BMI, TC, TG, HDL, LDL, FPG, LAP, and VAI are expressed as M (P_25_, P_75_), and smoking and alcohol consumption and history of hypertension are expressed as *N* (%).

### Prevalence of hypertension in oil workers with different individual characteristics

The prevalence of hypertension among oil workers was 10.1%, with 13.9% among men and 3.6% among women. The prevalence of hypertension among oil workers with different individual characteristics was statistically significant (*P* < 0.05) (Table 2).

**Table 2:** Prevalence of hypertension in oil workers with different individual characteristics.

Group	Hypertension (*N* = 220)	No hypertension (*N* = 1,966)	*t/* ${\chi ^2}$ */z*	*P*-value
Age (years)	49.23 $\pm$ 6.88	39.90 $\pm$ 9.53	−14.130	<0.001
Male (cases (%))	191 (86.8)	1,181 (60.1)	60.566	<0.001
Smoking (cases (%))	103 (46.8)	543 (27.6)	35.030	<0.001
Drinking (cases (%))	111 (50.5)	681 (34.6)	21.421	<0.001
BMI (kg/m^2^)	26.80 (24.50, 29.10)	24.10 (21.60, 26.60)	−10.652	<0.001
WC (cm)	93.94 $\pm$ 10.69	83.64 $\pm$ 11.58	−11.766	<0.001
WHtR	0.55 $\pm$ 0.06	0.49 $\pm$ 0.06	−13.19	<0.001
TG (mmol/L)	1.72 (1.24, 2.68)	1.18 (0.79, 1.84)	−8.541	<0.001
TC (mmol/L)	5.24 (4.58, 5.86)	5.09 (4.52, 5.77)	−1.420	0.156
HDL-C (mmol/L)	1.12 (0.98, 1.30)	1.26 (1.06, 1.50)	−6.727	<0.001
LDL-C (mmol/L)	2.92 (2.44, 3.46)	2.79 (2.31, 3.29)	−2.018	0.044
FPG (mmol/L)	5.55 (5.18, 6.12)	5.17 (4.88, 5.55)	−8.482	<0.001
Scr (μmol/L)	81.96 $\pm$ 35.87	72.82 $\pm$ 14.65	−7.161	0.001
LAP	49.62 (31.52, 82.88)	24.98 (11.84, 46.50)	−11.157	<0.001
VAI	2.17 (1.41, 3.35)	1.39 (0.83, 2.27)	−8.427	<0.001

**Note:**

Age, waist circumference (WC), WHtR, Scr are expressed with (x $\pm$ s), BMI, TC, TG, HDL, LDL, FPG, LAP, VAI are expressed with M (P_25_, P_75_), gender, smoking and alcohol consumption are expressed with *N* (%).

### Correlation analysis of hypertension prevalence and each body fat index in oil workers

In this study, Spearman rank correlation was used to analyze the correlation between the prevalence of hypertension and various body fat indicators such as WC, BMI, WHtR, LAP, and VAI in the study subjects (Table 3). The results suggested that the prevalence of hypertension in oil workers was positively associated with WC, BMI, WHtR, LAP, and VAI (*P* < 0.001).

**Table 3:** Correlation analysis of hypertension prevalence and each body fat index among oil workers.

	WC	BMI	WHtR	LAP	VAI
Male					
*r*	0.221	0.067	0.253	0.114	0.061
*P*	<0.001	0.014	<0.001	<0.001	0.024
Female					
*r*	0.176	0.188	0.195	0.191	0.132
*P*	<0.001	<0.001	<0.001	<0.001	<0.001

**Note:**

WC, waist circumference; BMI, body mass index; WHtR, waist height ratio; LAP, lipid accumulation product; VAI, visceral adiposity index; Spearman rank correlation was used for statistical analysis.

### Multifactorial logistic regression analysis of hypertension and LAP and VAI in oil workers

LAP and VAI quartiles were grouped (Q1, Q2, Q3, Q4) to establish a logistic regression model for hypertension in oil workers (Table 4). The results suggest that higher LAP and VAI among oil workers are independent risk factors for the development of hypertension (*P* < 0.001). After adjustment by model 2, the OR (95% CI) values of LAP in the third and fourth quartile groups were 3.71 (1.71–7.75) and 5.69 (2.72–11.89), respectively, using the lowest quartile group as a reference. The OR (95% CI) values were 2.63 (1.49 to 4.67) and 3.56 (2.03 to 6.23) in the third and fourth quartile groups compared to the lowest quartile group of VAI. The risk of hypertension increases gradually with the increase of LAP and VAI levels.

**Table 4:** Logistic regression analysis of hypertension prevalence and LAP and VAI in oil workers.

Independent variables	Model 1	*P*-value	Model 2	*P*-value
LAP (Q1)	1.00		1.00	
LAP (Q2)	3.96 (1.88–8.34)	<0.001	2.08 (0.96–4.50)	0.063
LAP (Q3)	9.08 (4.49–18.36)	<0.001	3.71 (1.77–7.75)	0.001
LAP (Q4)	14.23 (7.12–28.44)	<0.001	5.69 (2.72–11.89)	0.000
VAI (Q1)	1.00		1.00	
VAI (Q2)	2.24 (1.27–3.97)	0.006	1.84 (1.00–3.37)	0.049
VAI (Q3)	4.05 (2.38–6.92)	<0.001	2.63 (1.49–4.67)	0.001
VAI (Q4)	6.20 (3.69–10.41)	<0.001	3.56 (2.03–6.23)	0.000

**Note:**

Model 1, uncorrected; model 2, corrected for age, BMI; Scr, Scr-Serum creatinine; FPG, fasting plasma glucose.

### Predictive value of LAP, VAI, and LAP combined with VAI for hypertension in oil workers

**Figure 1:**
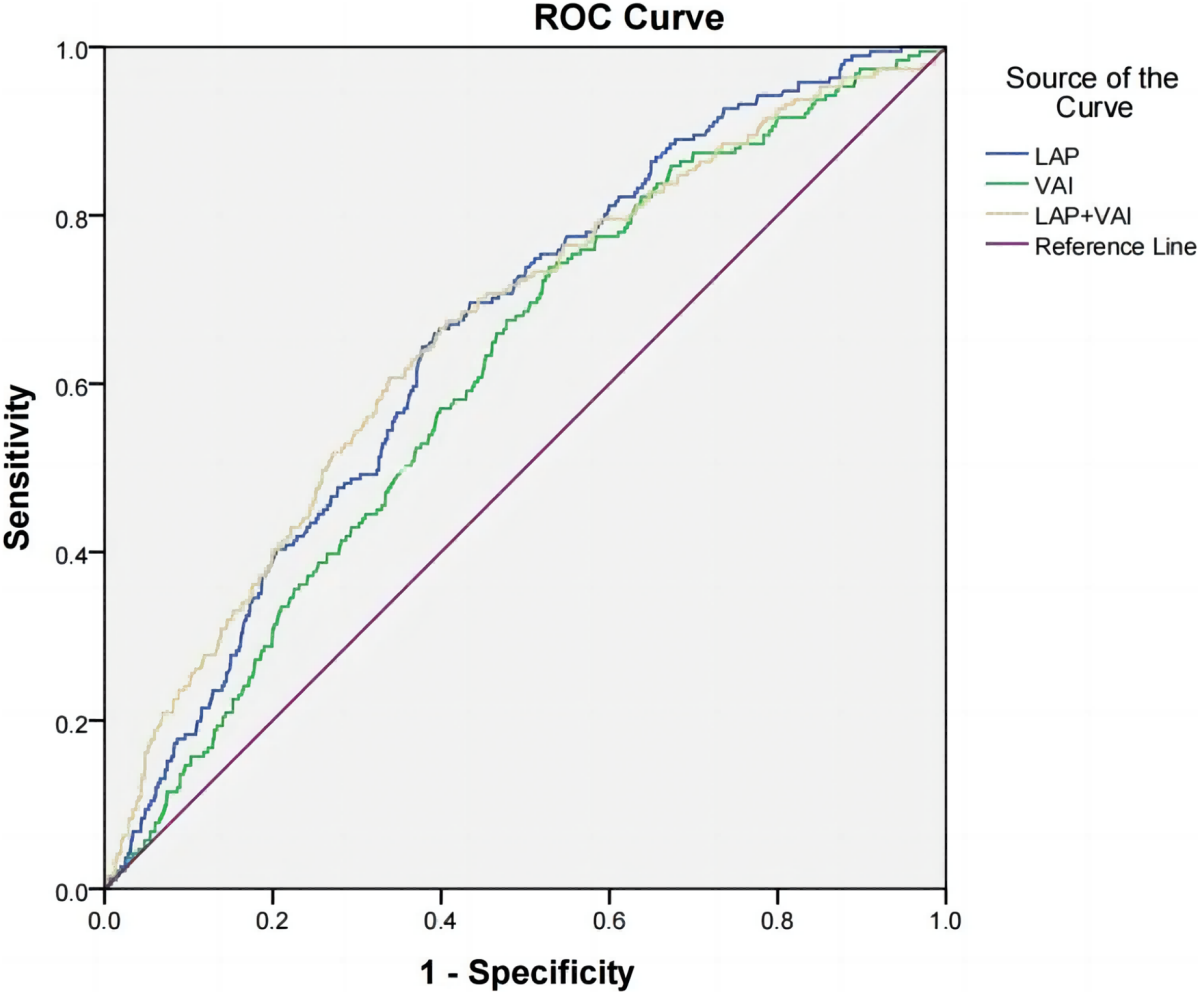
ROC curves of hypertension and LAP, VAI, and LAP combined with VAI in male oil workers.

**Figure 2:**
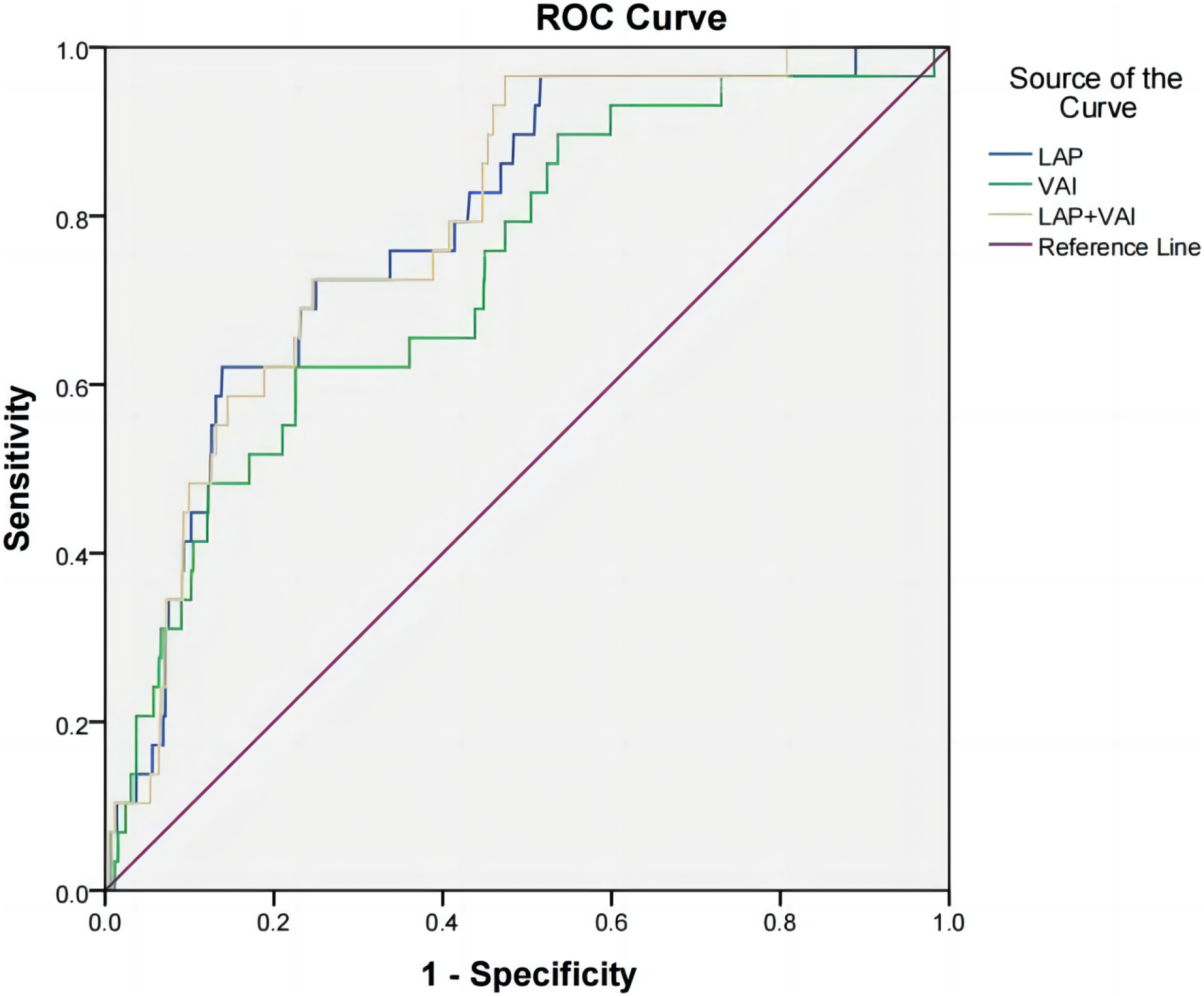
ROC curves of hypertension and LAP, VAI, and LAP combined with VAI in female oil workers.

According to the receiver operating characteristic curve (ROC), the area under curve (AUC) of LAP, VAI, and LAP combined with VAI were 0.658 (95% CI [0.619–0.696], *P* < 0.001), 0.614 (95% CI [0.574–0.654], *P* < 0.001), 0.661 (0.620–0.703, *P* < 0.001) in the male group; 0.787 (95% CI [0.710–0.865], *P* < 0.001) in the female group, 0.732 (95% CI [0.640–0.825], *P* < 0.001), 0.792 (0.719–0.864, *P* < 0.001). This suggests that LAP, VAI and LAP combined with VAI have some diagnostic value for hypertension prevalence. The cut-off values for LAP, VAI, and LAP combined with VAI were 43.245, 1.575, and 0.129 for the male group and 35.730, 1.759, and 0.025 for the female group, respectively (Table 5, Figs. 1 and 2).

**Table 5:** ROC curve analysis of LAP, VAI, LAP combined with VAI and hypertension in oil workers.

Variables	*AUC*	95% CI	*P*	Sensitivity	Specificity	Youden’s index	Truncation value
Male							
LAP	0.658	[0.619–0.696]	<0.001	0.660	0.608	0.268	43.245
VAI	0.614	[0.574–0.654]	<0.001	0.738	0.472	0.210	1.575
LAP+VAI	0.661	[0.620–0.703]	<0.001	0.675	0.593	0.268	0.129
Female							
LAP	0.787	[0.710–0.865]	<0.001	0.621	0.861	0.482	35.730
VAI	0.732	[0.640–0.825]	<0.001	0.621	0.775	0.395	1.759
LAP+VAI	0.792	[0.719–0.864]	<0.001	0.966	0.526	0.492	0.025

### Dose-response relationship between LAP, VAI index and hypertension prevalence

**Figure 3:**
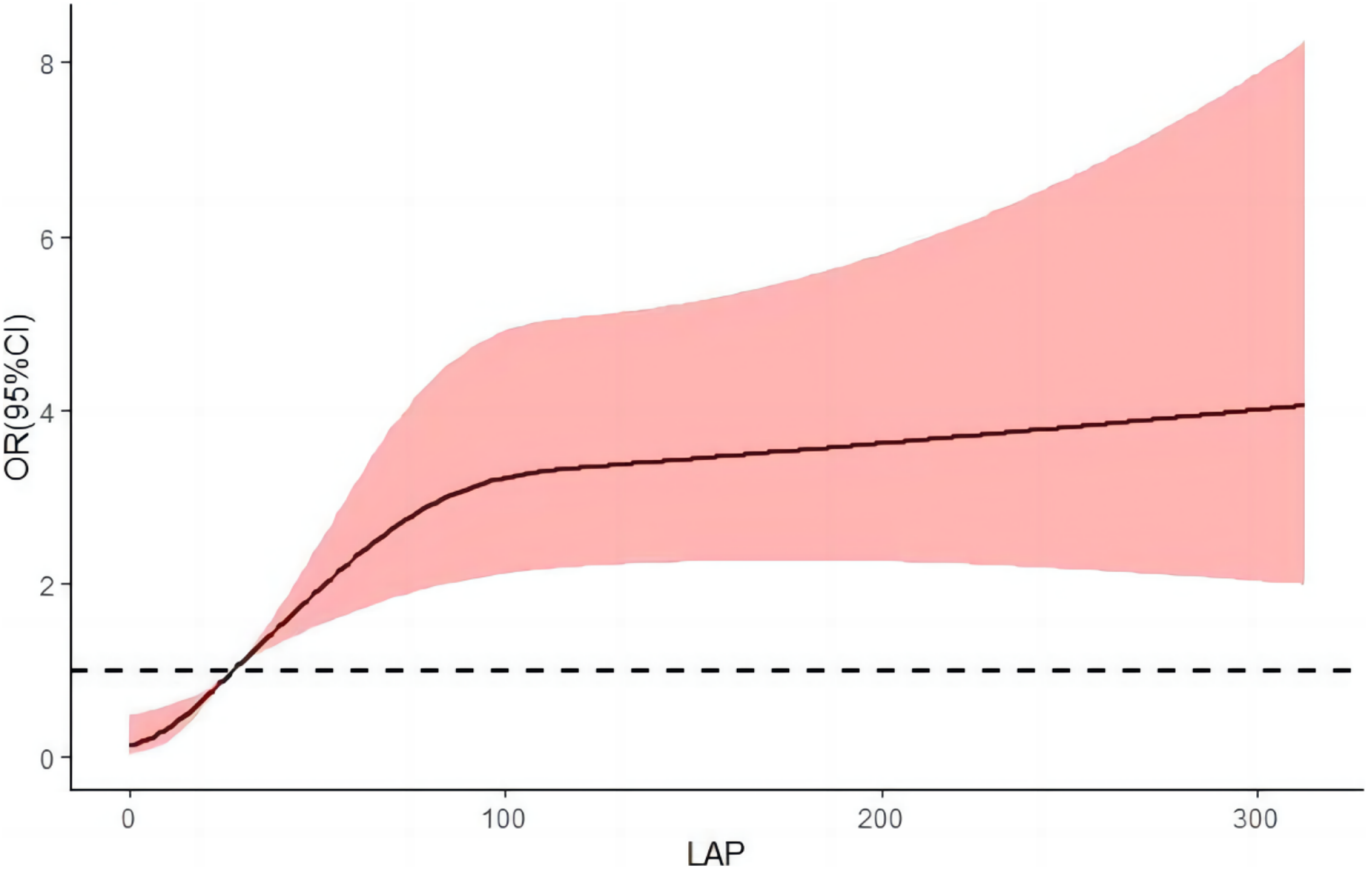
Dose-response relationship between LAP and hypertension prevalence.

**Figure 4:**
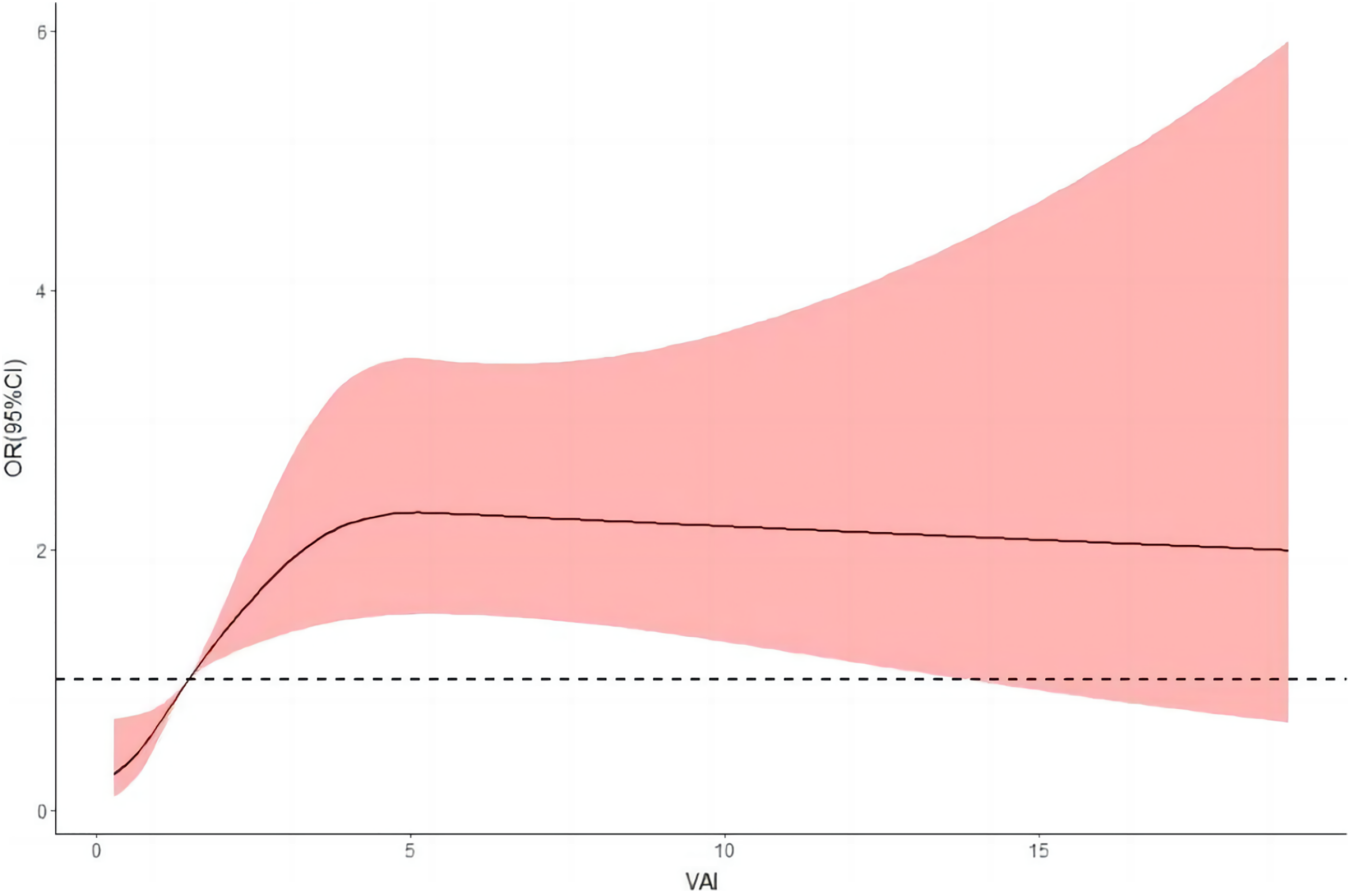
Dose-response relationship between VAI and hypertension prevalence.

After adjusting for relevant confounders, the restricted cubic spline results illustrate a nonlinear dose-response relationship between LAP, VAI, and risk of hypertension (*P* < 0.001 for overall trend and *P* < 0.001 for nonlinearity). The risk of hypertension increased rapidly before a VAI index of four and then began to decrease gradually; as the LAP index increased, the risk of hypertension increased, and as the LAP continued to increase above 80, the risk of hypertension began to decrease, but the decrease was small (Figs. 3 and 4).

## Discussion

This survey showed that the prevalence of hypertension was 10.1%, with 13.9% in men and 3.6% in women. The prevalence of hypertension with different individual characteristics was statistically significant (*P* < 0.05). Hypertension is caused by many factors, among which obesity is closely related to hypertension (Garrison et al., 1987). And visceral fat has an important role in the development of hypertension. However, the traditional obesity index (BMI) has limitations in distinguishing the difference between subcutaneous and visceral fat. LAP is considered to be a relatively accurate reflection of the degree of lipid accumulation and visceral fat content in humans and is suitable for comprehensive measures of obesity in epidemiological survey studies (Zhong et al., 2016; Joshi et al., 2016). VAI is a novel body fat index based on a series of metabolic parameters proposed by Amato et al. (2010) and is a good indicator to assess visceral adiposity function (Štěpánek et al., 2019). It has advantages such as simple calculation method, low cost, and no ionizing radiation. However, existing correlations between LAP, VAI and hypertension have only been performed in the general population, with a lack of relevant studies in occupational populations.

In this study, a survey of oil workers found that hypertension prevalence was positively associated with lipid accumulation product and visceral adiposity index. After correcting for relevant confounders, the risk of hypertension increased with increasing levels of LAP and VAI. This is consistent with the findings of Wang et al. (2018) who showed a significant association between LAP and hypertension in a cross-sectional study of people in rural China, and Kyrou et al. (2018) who showed that LAP was positively associated with the incidence of cardiovascular disease in adult whites and was a better predictor of long-term cardiovascular disease risk than common indicators of obesity. A study by Yang et al. (2020) demonstrated that VAI was positively associated with hypertension in the Chinese adult population and could be used as a predictor of hypertension risk. The mechanism of interaction between visceral obesity and hypertension is complex and may be related to reduced levels of adiponectin and secretion of more leptin in viscerally obese populations, and preliminary evidence suggests that activation of the adiponectin-angiotensin system is associated with hypertension in models of visceral obesity (Kotchen, 2010).

Considering the important gender differences in human fat distribution and lipid metabolism (Zhou et al., 2020), we did a stratified analysis. The results showed that men had higher levels of VAI and LAP than women. This gender difference may be influenced by obesity and sex hormones. Compared to women, men have more risk factors for obesity, such as smoking and alcohol consumption, excessive intake of ultra-processed foods, especially meat ready-to-eat products, and sugary drinks. Further analysis of the predictive value of LAP and VAI on the prevalence of hypertension revealed that the predictive value of LAP, VAI and the combination of the two on the prevalence of hypertension was higher in women than in men, probably due to anatomical, physiological, sex hormone levels and metabolic differences, and different fat distribution in men and women, the exact reason for this gender difference is unclear, and further studies are needed to confirm the gender difference in the prediction of the risk of hypertension by VAI and LAP. According to a survey study (Feng et al., 2016), the percentage increase in the population attributable risk for the development of hypertension due to overweight/obesity in women has been greater in recent years, and interventions for the female population should be strengthened. Lee et al. (2011) studied 1,777 population in Bengbu, China showed that the cut point for LAP to predict hypertension was 40.60 in men and 29.14 in women. The present study showed that the best critical values of LAP and VAI to predict hypertension were 43.245 and 1.575 for men and 35.730 and 1.759 for women, respectively, which were not completely consistent with the above-mentioned studies, probably due to differences in dietary habits and population characteristics in different regions. Xinjiang is characterized by a multi-ethnic gathering and has a special lifestyle and diet, which leads to an increased incidence of chronic diseases such as hypertension.

In Figs. 3 and 4, we used restricted cubic splines to flexibly model and visualize the nonlinear dose-response relationship between predicted LAP and VAI and risk of hypertension in oil workers. This is one of the highlights of our current study. Our findings revealed that there was not a simple linear variation between them, and the RCS plot clearly reflected the overall trend of the LAP and VAI indices with the risk of developing hypertension. To the best of our knowledge, no relevant studies have been conducted to reflect their relationship by plotting RCS curves. We believe that the mechanism of action of this nonlinear dose-response relationship between visceral obesity and hypertension deserves further exploration and study in the future.

## Conclusions

This study found that LAP and VAI are positively correlated with hypertension, and LAP and VAI have certain predictive value for the occurrence of hypertension. Compared with the typical body fat index, LAP and VAI are simple, fast and low cost as assessment indexes for visceral obesity, which can be widely used at the grassroots level and provide guidance for lifestyle interventions in primary hypertensive patients to control body weight, pay attention to low-fat diet and reduce visceral fat accumulation, which is beneficial to the prevention and control of hypertension.

### Limitations

This study has the following limitations. Firstly, the present study is a cross-sectional study and the causal relationship between LAP, VAI and hypertension could not be analyzed. Secondly, the sample of this study was a single-center sample from the occupational population in Xinjiang, and there are some limitations of extrapolation. Future longitudinal epidemiological studies with large samples from different regions and populations are still needed.

## Supplemental Information

10.7717/peerj.15273/supp-1 Supplemental Information 1 Data.

## Additional Information and Declarations

### Data Availability

The following information was supplied regarding data availability: The raw measurements are available in the Supplemental File.
